# Ozone, and Its Connection with Epidemic Diseases

**Published:** 1849-08

**Authors:** 


*Ozone, and its Connection with Epidemic Diseases.* — Ozone, to which influenza is ascribed by Schonbein, and cholera by some of our western brethren, has been variously described and defined. It has been imagined to be a combination of nitrogen and oxygen in some new proportion; or a new combination of oxygen and hydrogen. Spengler is quoted in the *Lon. Med. Gaz.* for Nov. 1848, as saying that it is "formed in the air, by the decomposition of its water through disturbance of its electrical equilibrium." Professor Draper, of the New York University, regards it as the active state of oxygen, or oxygen rendered active by electricity: This opinion, which is clear and intelligible, seems to be proved to be true, also, by the experiment of passing a current of electric fluid through pure oxygen; ozone is thus obtained, having a sulphurous odor, setting fire to phosphorous, and irritating the nostril, as in catarrh.

The test of its presence is a bit of paper dipped in a solution of iodide of potassium, and then in one of starch. The oxygen of common air acts slowly on it, and produces gradual change, and coloration. Ozone and ozonized air will occasion them to act promptly on each other, producing a dark blue color.

The description given by Spengler of its mode of production is not easily understood; he goes on as vaguely to add:—"Hence its peculiar pungent sulphurous, phosphorus odor." Whence? From the electrical decomposition of water. This gives us nothing that we know of but oxygen and hydrogen, which in their ordinary states, have no such odor. He goes on to say, that "sulphuric, probably telluric, selenic, and phosphoric acids destroys it. Vapors of ether, or alcohol, or olefiant gas prevent its development."

Mr. Moffat,* from the results of his experiments and observations, comes to the conclusion that the presence of ozone in large quantities in the atmosphere is invariably attended with catarrh and mucous diarrhœa. Dr. Golding Bird,† at a meeting of the Medical Society of London, held in October last, while the subject of cholera was under discussion, said that he had remarked and noticed it as a somewhat curious fact, that of late ozone, or peroxide of hydrogen, had been found in the atmosphere. Dr. Parkin, an English physician, residing in Spain, says he has used charcoal with great success in the treatment of cholera; he used it in doses from a tea-spoonful to a table-spoonful in water, every two or three hours. Dr. Spengler, from his observations, has been led to believe that sulphur or sulphurous gases must be an antidote to the poisonous influences of ozone. Dr. Bird aud Professor Herrick, of Chicago, have detected the presence of ozone in the atmosphere of Chicago during the present

*British Association.—Athenæum Report. †London Med. Gaz. for Oct.

epidemic, and both speak decidedly favorable of three or four grain doses of sulphur combined with charcoal. Professor Herrick says, "that all premonitory symptoms, such as pain, a sense of fulness, unnatural movements, slight diarrhœa, &c. have uniformly yielded to this remedy" (?Ed.) In this city no such success has followed its use. To the pathologist the inquiry into the nature and influence of ozone has, indeed, become truly interesting.—*N. Y. Journal of Medicine—Editorial.*

